# A GIS-Based Artificial Neural Network Model for Flood Susceptibility Assessment

**DOI:** 10.3390/ijerph18031072

**Published:** 2021-01-26

**Authors:** Nanda Khoirunisa, Cheng-Yu Ku, Chih-Yu Liu

**Affiliations:** Department of Harbor and River Engineering, National Taiwan Ocean University, Keelung City 20224, Taiwan; 10786013@mail.ntou.edu.tw

**Keywords:** geographic information system, back-propagation neural network, rainfall, historical flood, prediction

## Abstract

This article presents a geographic information system (GIS)-based artificial neural network (GANN) model for flood susceptibility assessment of Keelung City, Taiwan. Various factors, including elevation, slope angle, slope aspect, flow accumulation, flow direction, topographic wetness index (TWI), drainage density, rainfall, and normalized difference vegetation index, were generated using a digital elevation model and LANDSAT 8 imagery. Historical flood data from 2015 to 2019, including 307 flood events, were adopted for a comparison of flood susceptibility. Using these factors, the GANN model, based on the back-propagation neural network (BPNN), was employed to provide flood susceptibility. The validation results indicate that a satisfactory result, with a correlation coefficient of 0.814, was obtained. A comparison of the GANN model with those from the SOBEK model was conducted. The comparative results demonstrated that the proposed method can provide good accuracy in predicting flood susceptibility. The results of flood susceptibility are categorized into five classes: Very low, low, moderate, high, and very high, with coverage areas of 60.5%, 27.4%, 8.6%, 2.5%, and 1%, respectively. The results demonstrate that nearly 3.5% of the study area, including the core district of the city and an exceedingly populated area including the financial center of the city, can be categorized as high to very high flood susceptibility zones.

## 1. Introduction

Coastal areas are vulnerable to climate change, particularly sea-level rises and floods related to extreme rainfall [1,2]. Taiwan is an island which is prone to flood disasters triggered by heavy rainfall and typhoons every year. In Taiwan, extreme weather conditions, such as heavy precipitation and typhoons generated by climate change, strengthen the phenomenon of flood disasters [3]. Keelung City, one of the coastal cities in Northern Taiwan, has become highly urbanized and densely populated in recent years [4]. Flooding events have frequently occurred in the past because typhoons and rainstorms typically sweep over the upstream basins between May and October; this trend is expected to increase in the future [5,6,7].

Various approaches have been proposed to evaluate flood disaster risk based on the susceptibility of the system and hydrology [8,9,10]. The artificial neural network (ANN) is one of the most implemented machine-learning techniques in engineering risk assessment [11]. The ANN model is a network of machine learning that is based on the human brain [12]. Nowadays, ANNs and computational intelligence (CI) methods are often used for flood disaster modeling [13]. Machine-learning technologies have been applied for analysis of flood susceptibility assessment, including logistic regression, radial basis function (RBF) neural network, and support vector machine (SVM) [14,15,16]. For logistic regression, this algorithm is easier to implement and interpret, and more efficient to train [14]. However, it may lead to overfitting. For the RBF neural network, it performs more robustly and tolerantly than conventional neural networks, especially when dealing with noisy data [15]. For SVMs, it is more effective in high-dimensional spaces [16]. Despite the success of the above machine-learning technologies as effective numerical tools for engineering risk assessment, there is still growing interest in the development of a more accurate predictive risk model. The analysis of the spatial distribution of flood disaster risk plays an important role, especially regarding disasters occurring along coasts and rivers [17,18,19]. Spatial analysis is applied to define the relationship between flood factors in hazards, vulnerability, and risk through the map, without focusing on complex hydrological modeling [20]. For flood analysis, studies using geographic information system (GIS) technologies, remote sensing, and numerical models, and adopting an artificial neural network approach, have been widely used around the world [21,22,23,24,25,26].

This article presents a GIS-based artificial neural network (GANN) model for the flood susceptibility assessment of Keelung City, Taiwan. Various factors, including elevation, slope angle, slope aspect, flow accumulation, flow direction, topographic wetness index (TWI), drainage density, rainfall, and normalized difference vegetation index, were generated using a digital elevation model and LANDSAT 8 imagery. Historical flood data from 2015 to 2019, including 307 flood events, were adopted for a comparison of flood susceptibility. Using these factors, the GANN model, based on the back-propagation neural network (BPNN), was employed to assess flood susceptibility. The main contribution of this work is that the proposed method, based on ANN and GIS, may improve the ability to establish further precise flood models, and present the results in a spatial environment. The advantages of the GIS spatial analysis capability were integrated into the artificial neural network model. This work is organized as follows. In Section 2, the methodology is introduced. Results are presented in Section 3. Discussions are presented in Section 4, and key findings of this pioneering work are summarized in this section. Conclusions are made in Section 5.

## 2. Materials and Methods 

### 2.1. Description of the Study Area

**Figure 1:**
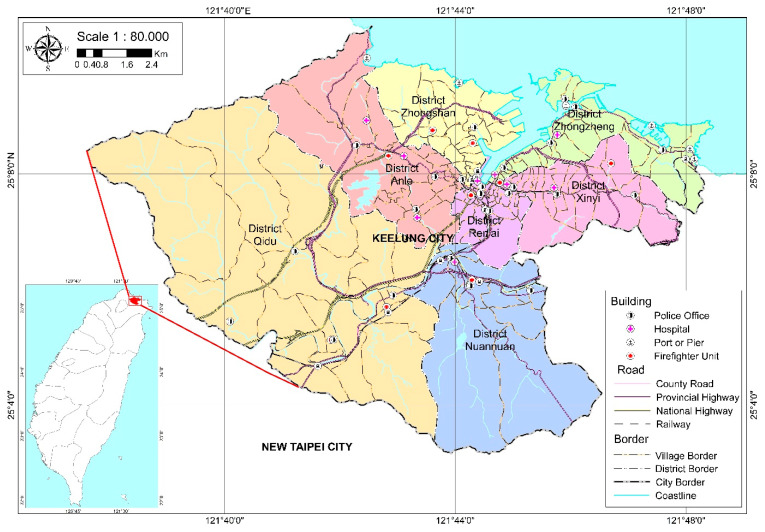
Administration map of Keelung City, Taiwan.

The research area was Keelung City, which is located in the northeastern part of Taiwan. The city area covers 132.7589 km^2^, and is divided into 7 districts and 157 villages, as shown in Figure 1. The city is also known as the rainy port for its high frequency of rain, with a yearly rainfall average upwards of 3700 mm. Keelung City is one of the major coastal cities in Northern Taiwan which has become highly urbanized and densely populated in the last few years [4].

### 2.2. Preparation of Data and Geospatial Layer

**Figure 2:**
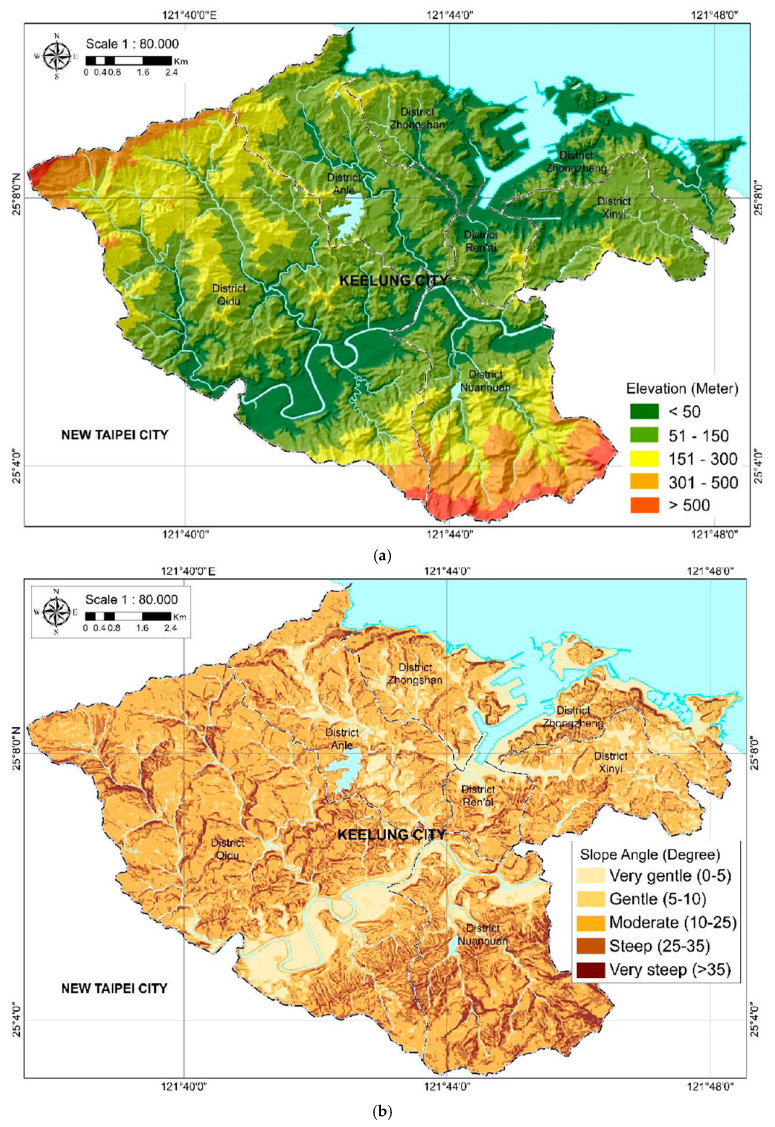

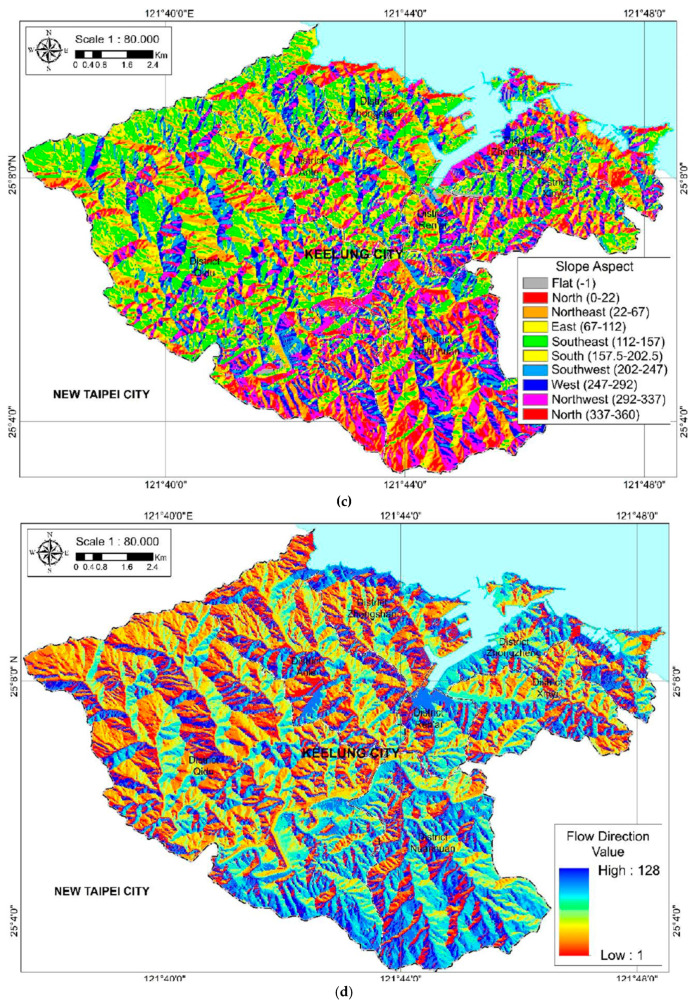

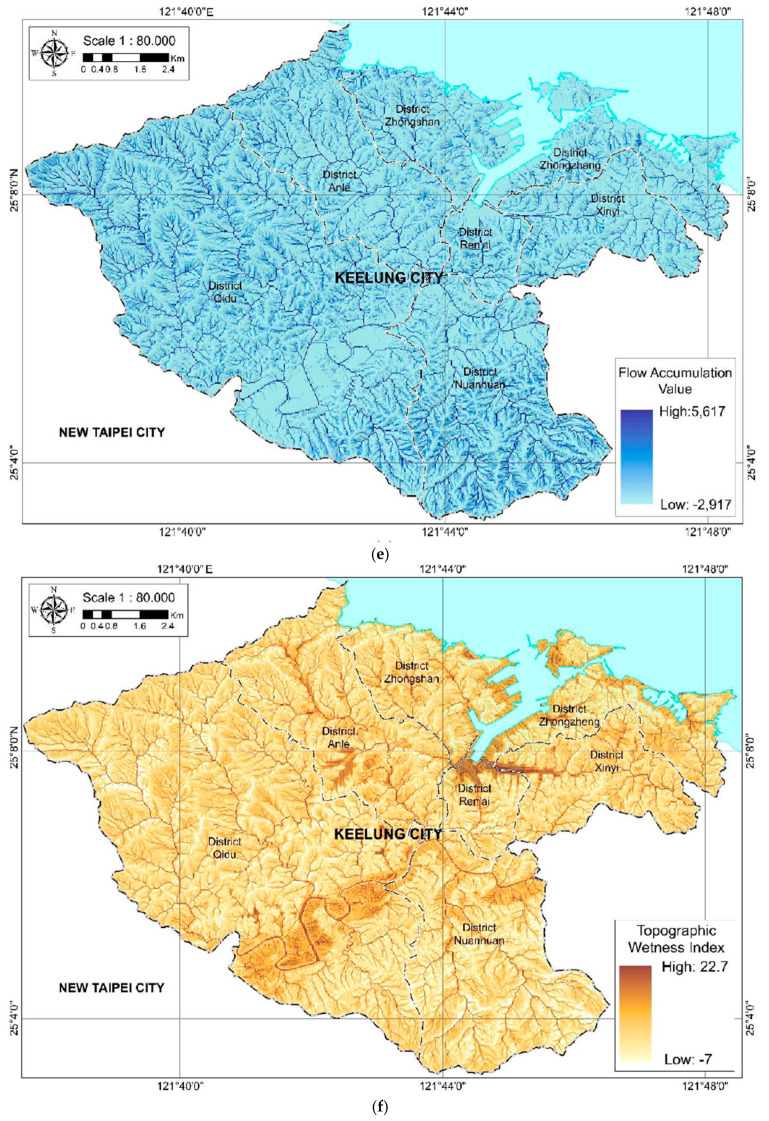

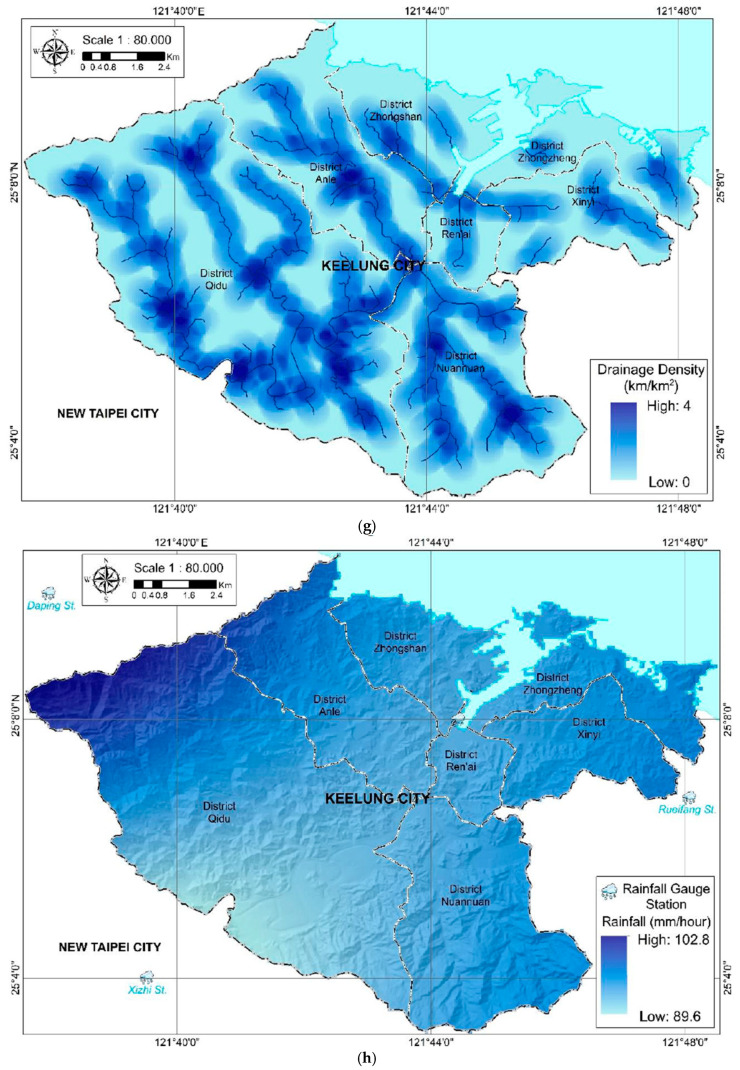

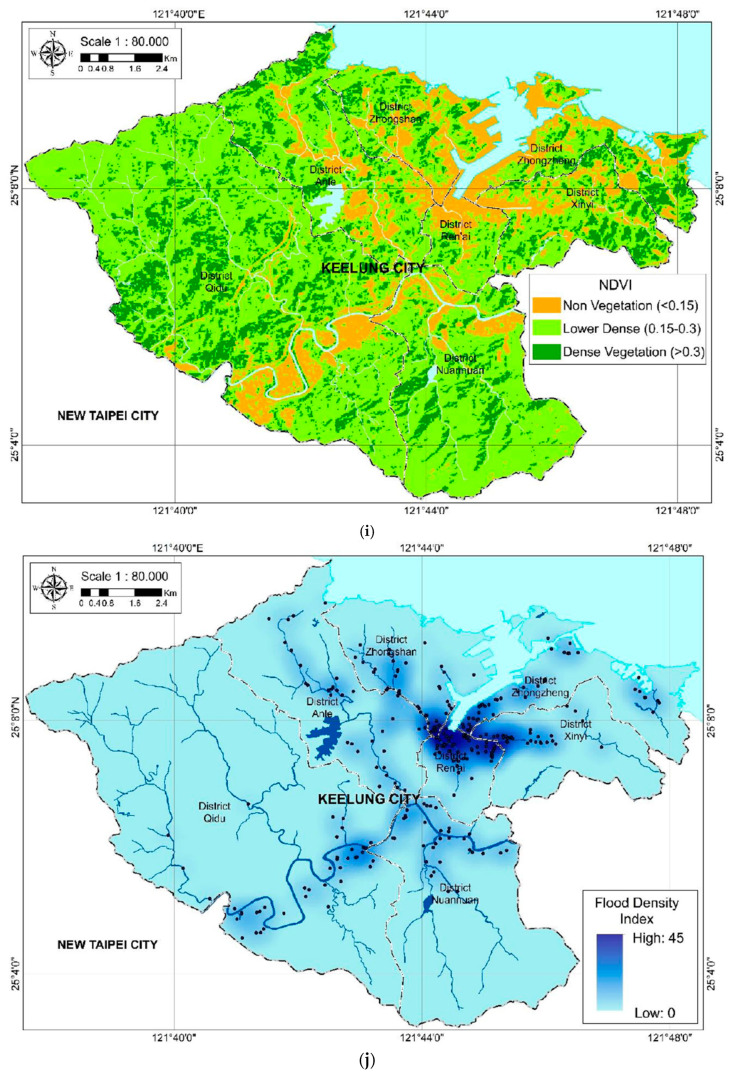
Geomorphologic area of Keelung City and relevant factors: (**a**) Elevation; (**b**) slope; (**c**) slope aspect; (**d**) flow direction; (**e**) flow accumulation; (**f**) TWI; (**g**) drainage density; (**h**) rainfall interpolation; (**i**) normalized difference vegetation index (NDVI); and (**j**) historical flood data.

Table 1 depicts the source data of the factors. The geomorphologic area of Keelung City and the relevant factors are shown in Figure 2. Factors including elevation, slope angle, slope aspect, flow accumulation, flow direction, and TWI were generated from the digital elevation model (DEM), with a resolution of 20 m. The LANDSAT 8 imagery, with a resolution of 30 m, from the United States Geological Survey, was used to generate the normalized difference vegetation index. Detailed descriptions of the factors are as follows.

#### 2.2.1. Elevation

The elevation map is a representation of altitude, with ranges between 0 and 783 m. Elevation is another frequently used parameter and one of the key factors controlling the floods of an area [21,22]. Flood disasters tend to occur in low altitude areas, compared with landslides that have a tendency to happen at higher altitudes [23]. Generally, water continually flows from higher elevations to lower elevation areas. 

#### 2.2.2. Slope

This topographic factor is fundamental in hydrological studies. The relationship with rainfall is likely that the slope is directly influenced by the infiltration of rainfall [24]. The slope of an area and the surface flow velocity could have a positive correlation. The slope is also very closely connected to the flow of runoff directly toward downstream; a higher magnitude of slope in an area might accelerate precipitation-related runoff. The surface runoff increases significantly as the gradient increases; consequently, the infiltration decreases. As an outcome of this, regions with a sudden decrease in the slope have a higher probability of flooding as a massive volume of water becomes stationary, which causes a severe flood disaster situation.

#### 2.2.3. Slope Aspect

This aspect recognizes the downslope direction, and is also thought of as the slope orientation [24]. The aspect of the slope presents the steepness of the surface, and is represented by three groups based on color brightness or saturation. The pixel values in the output aspect–slope raster represent a combination of aspect and slope. The aspect is one of the significant factors in producing flood susceptibility maps [25]. 

#### 2.2.4. Flow Direction

The hydrologic characteristics of a surface are the capacity to establish the flow direction of each raster cell. The flow direction is a grid whose value indicates the direction of flow for every cell to its steepest downslope neighbor in the DEM [26].

#### 2.2.5. Flow Accumulation

The flow accumulation tool generates accumulated flow as the accumulated weight of all cells flowing into each downslope cell in the output raster. The results of flow accumulation can be used to create a stream network by applying a threshold value to select cells with a high accumulated flow.

#### 2.2.6. Topographic Wetness Index 

The TWI is a physical representation of flood inundation areas, which is an important component of a river catchment. The TWI of a catchment indicates two types of measurements: Flat lands and hydrographic positions. The TWI is commonly used to quantify topographic control of hydrological processes. It is expressed as

(1) $$TWI\;=\;\ln\left(\frac{\alpha }{\tan\beta }\right),$$

where α is the cumulative upslope contribution area draining through a point (per unit contour length), and tanβ is the slope angle at the point. It affects the spatial distribution of soil moisture, and the groundwater flow often follows the surface topography. In this study, TWI is considered another contributing factor. Areas with a high wetness index occur where there is a combination of low slope and high flow accumulation and, therefore, may indicate locations that are at greater flood risk [27].

#### 2.2.7. Drainage Density

The calculation of the drainage system raster was done by using the line density method, with rivers (polyline) as the main input data. The unit of calculated density was the length per unit of area. A higher probability of flooding is strongly associated with higher drainage density, as it represents greater surface runoff. The drainage density map of Keelung City was calculated from the drainage network map using line density tool in the ArcGIS, and ranges from 0 to 4 km/km^2^. 

#### 2.2.8. Rainfall Interpolation

Rainfall-induced flooding is associated with tropical storms, hurricanes, tropical depressions, and west trade winds that directly strike the windward side of the highlands. A large number of previous studies in the literature have established a relationship between the rainfall and the flood occurrence of an area [28,29,30]. Preparation of a rainfall map in this study used the pixel-based highest hourly rainfall data of several decades, spread around four rain gauge stations around Keelung City, with the Kriging interpolation method [31]. The rainfall data were collected from Keelung station, Xizhi station, Ruifang station, and Daping station through the Central Weather Bureau (CWB) of Taiwan. 

#### 2.2.9. Normalized Difference Vegetation Index (NDVI)

The NDVI is one of the most extensively adopted vegetation indexes using satellite imagery, and for monitoring of global vegetation cover [32]. The NDVI, developed by Rouse in 1973, is used to monitor vegetation health, and to compare outputs across sensors with slightly different specifications [33]. The NDVI equation is defined as

(2) $$\mathrm{NDVI}\;=\;\frac{NIR-RED}{NIR+RED},$$

where *NIR* is the reflection in the near-infrared spectrum and *RED* is the reflection in the red range of the spectrum. *NIR* and *RED* represent near-infrared (λ ~ 0.8 μm) regions of the spectrum and surface reflectance averaged over the visible spectrum (λ ~ 0.6 μm), respectively. The NDVI data source for this layer was the LANDSAT 8 OLI/TIRS C1 Level 1, to match the multispectral bands captured on 13 March 2018 and the area coverage of the study area on path 117 and row 43. The satellite imagery was rendered as NDVI, and colorized for use in the visualization analysis. Specifically, the source of the NDVI layer is the metadata of the imagery, which shows land cloud cover of only 0.8% and scene cloud cover of 1.91%. The NDVI response indicates the effective flood extent as an influential factor for the strength and capacity of an area against flood hazards. 

#### 2.2.10. Historical Flood Density

**Figure 3:**
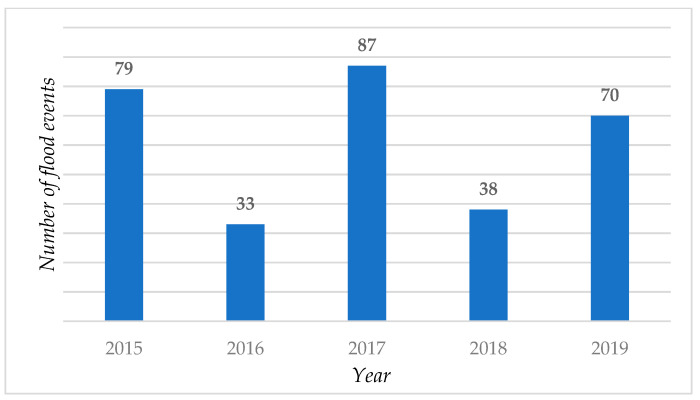
Historical flood data in Keelung City, Taiwan.

Information on the historical floods in Keelung City was collected from the Emergency Management Information Cloud (EMIC) of Taiwan from 2015 to 2019, and comprised 307 events. The number of events each year is shown in Figure 3. The flood history, also known as the disaster experience, assumes that such areas have higher adaption ability, but also a high probability of flood occurrence in the future [3,34]. The density of point features in the raster cell unit is generated using the kernel density to fit a smoothly tapered surface to each point of the flood history. The kernel density tool in ArcGIS calculates the density of features in a neighborhood around the features, to find the spatial analysis and previous flood history of the area. By calculating the values of all the kernel surfaces, the density of each output raster cell feature is determined. 

### 2.3. Artificial Neural Network

**Figure 4:**
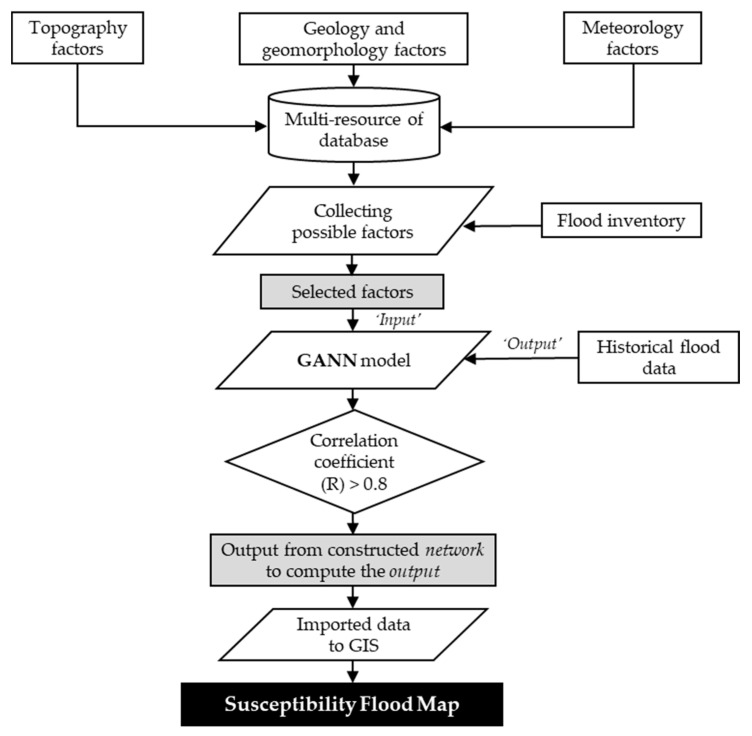
Flow chart of the study.

The ANN is considered a quantitative black-box approach that tries to simulate the functional human biological nervous system [12]. Moreover, the environment nonlinearity analysis and the forecast can be studied by applying this effective and affordable machine-learning tool. Even though the ANN has also been effectively applied to flood analysis in previous investigations [21,28], the GANN model is still rarely used for flood susceptibility assessment. Furthermore, the ANN has been considered as an alternative to physically-based models due to its simplicity regarding the minimum requirements for collecting detailed data [35]. The schematic and conceptual flowchart of this study is depicted in Figure 4. From Figure 4, three major factors, including topography factors, geology and geomorphology factors, and meteorology factors, were considered as a multi-resource aspect of the database in this study. Various factors, including elevation, slope angle, slope aspect, flow accumulation, flow direction, TWI, drainage density, rainfall, and NDVI, were generated using the digital elevation model, as well as LANDSAT 8 imagery. Historical flood data from 2015 to 2019, including 307 flood events, were adopted for the comparison of flood susceptibility. After collecting the possible factors, the proposed GANN model, based on the BPNN, was employed to assess the flood susceptibility. Finally, the accuracy of proposed GANN model predictions was evaluated by calculating the correlation coefficient.

The GANN was employed to assess the flood susceptibility of Keelung City. This research focuses on a BPNN [36,37]. A typical algorithm of artificial neurons comprises three layers: Input, hidden, and output layers. The BPNN algorithm of feedforward shows an essential feature of the training phase, and the progress result is expressed as follows:

(3) $$y_{i\;}=\;F(X_{j})\;=\;\left(W_{oj}+\sum_{i=1}^{I}W_{ij}x_{i}\right),$$

(4) $$Z_{k}=F(Y_{k})=\left(W_{ok}+\sum_{j=1}^{J}W_{kj}y_{i}\right),$$

where $x_{i}$*,* $y_{i}$, and $Z_{k}$ indicate the input, hidden, and output layers, respectively. The bias weights for setting the threshold values are represented as $W_{oj}$ and $W_{ok}$. Meanwhile, $X_{j}$ and $Y_{k}$ indicate temporarily calculated results before using the activation function, and F is the activation function applied in the hidden and output layers. The F value ranges from 0 to 1. In this study, we adopted the hyperbolic tangent sigmoid function. A sigmoid function is a mathematical function with a characteristic S-shape curve or sigmoid curve. The sigmoid activation function is a widely used activation function for neural networks [38,39]. The positive input value to the function is transformed into a value between 0.0 and 1.0. Inputs that are much larger than 1.0 are transformed to the value 1.0; similarly, values much smaller than 0.0 are snapped to 0.0. The shape of the function for all possible inputs is an S-shape from zero up through 0.5 to 1.0. Since the probability of anything exists only between the range of 0.0 and 1.0, the sigmoid activation function is adopted as the activation function for the proposed neural networks. Thus, the output $y_{i}$ and $Z_{k}$ can be expressed as

(5) $$y_{i}=F(X_{j})=F\left(\frac{1}{1+e^{-X_{j}}}\right),$$

(6) $$Z_{k}=F(Y_{k})=F\left(\frac{1}{1+e^{-Y_{k}}}\right).$$

For the error back-propagation weight training, the error function can be established as

(7) $$E=\frac{1}{2}\sum_{k=1}^{K}\varepsilon_{k}^{2}=\frac{1}{2}\sum_{k=1}^{K}{\left(t_{k}-z_{k}\right)}^{2},$$

where $t_{k}$ and $\varepsilon_{k}$ are the target value and error in each output node, respectively. The goal is to minimize E, the error between the actual output values of the network. The weight adjustment in the link between the hidden and output layers can be expressed as

(8) $$\Delta w_{jk\;}=\;\eta \;\times \;y_{j}\times \;\delta_{k},$$

where η is the learning rate, with values ranging between 0 and 1. The learning rate values correlate with the speed of convergence of the network of the BPNN. Conversely, a learning rate that is overly large can lead to a widely oscillating network. The new weight herein is updated by the following equation:

(9) $$w_{jk}\left(n+1\right)=w_{jk}\left(n\right)+\Delta w_{jk}(n),$$

where n is the number of iterations in the network. Similarly, the error gradient in links between the input and hidden layers can be derived from the partial derivative with respect to $w_{ij}$:

(10) $$\frac{\partial E}{\partial w_{ij}}=\left[\sum_{k=1}^{K}\frac{\partial E}{\partial z_{k}}\frac{\partial z}{\partial Y_{k}}\frac{\partial_{k}}{\partial y_{j}}\right]\times \left(\frac{\partial y_{j}}{\partial X_{j}}\right)\times \left(\frac{\partial X_{j}}{\partial w_{ij}}\right)=-\Delta_{j}x_{i},$$

(11) $$\Delta_{j}=F^{\prime }(X_{j})\sum_{k=1}^{K}\delta_{k}w_{jk}.$$

The new weight in the hidden and input links can be regenerated as

(12) $$\Delta w_{ij\;}=\;\eta \;\times \;x_{i}\;\times \;\Delta_{j\;},$$

(13) $$w_{ij}\left(n+1\right)=w_{ij}\left(1\right)+\Delta w_{ij}(n).$$

To evaluate the prediction performance of the proposed GANN model, the correlation coefficient (R) is utilized and expressed as

(14) $$R=\frac{\sum_{i=1}^{n}\left(t_{i}-\bar{t}\right)(o_{i}-\bar{o})}{\sqrt{\sum_{i=1}^{n}{\left(t_{i}-\bar{t}\right)}^{2}\sum_{i=1}^{n}{\left(o_{i}-\bar{o}\right)}^{2}}},$$

where $t_{i}$ represents the target value and $o_{i}$ is the output value. Meanwhile, $\bar{t}$ and $\bar{o}$ are the average values of the target and output values, respectively. 

**Figure 5:**
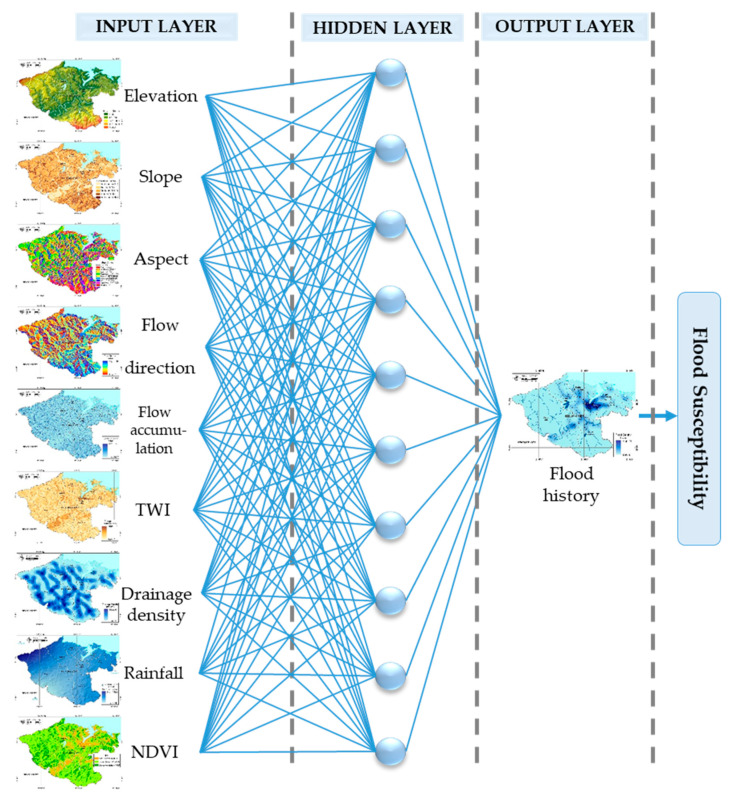
Structure of a typical three-layer model for flood susceptibility.

The BPNN algorithm is used in the feedforward GANN, and the structure of the neural network model is displayed in Figure 5. The selected input, containing nine quantitative input variables and one quantitative output variable, includes elevation, slope, TWI, rainfall, NDVI, flow accumulation, drainage density, flow direction, and slope aspect, as shown in Figure 5. The flood history data from 2015 to 2019 in Keelung City were the only output of this model. All the input data in the GANN model were normalized in the range 0–1, with the initial weights automatically assigned to random values. 

## 3. Results

### 3.1. Results of the Artificial Neural Network Model

The network architecture was designed to determine the flood susceptibility of Keelung City; an output layer of the flood history was required for the architecture of the model. This was used to evaluate the prediction performance of the train validation and test the model using the mean squared normalized error performance function, which measures the network’s performance function according to the mean squared error (MSE).

The GANN study needed to classify the variation data in the training process. Kia et al. [21] indicated that 60% should be used for training, 20% for validation, and 20% as testing data, while Aziz et al. [29] and Latt and Wittenberg [40] used a combination of 80% and 20% for training and the testing process, respectively. In this study, the flood data were divided into three groups: 70% for training the network, 15% for validating the model, and 15% for testing the data to completely independently test the network generalization. The chosen training algorithm of this research was Levenberg–Marquardt (LM), which has the fastest training compared to Bayesian regulation back-propagation. This latter method takes longer, but may be better for challenging problems, while scaled conjugate gradient back-propagation is suitable for low-memory situations and was not used here [41]. The LM algorithm was selected as the training function, which combines the Gauss–Newton method and the gradient descent method. The LM algorithm was used to solve non-linear least squares problems and for its fault tolerance and fast convergence ability.

The number of hidden layers, along with neurons inside the model, is frequently defined by trial and error. The number of neurons in the output layers is fixed by the application, and is represented by the class being processed. The GANN model of flood susceptibility used 10 hidden layers, as at that point the model starts to reach the minimum requirement for the correlation coefficient, according to some research [8,28,42]. 

**Figure 6:**
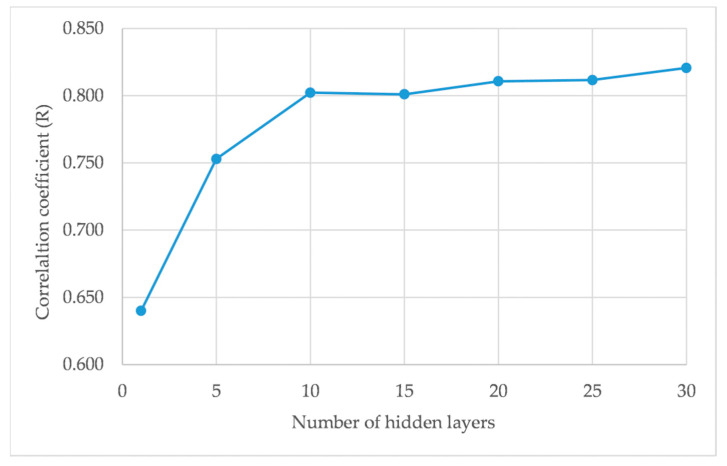
Number of hidden layers versus the value of R.

Figure 6 shows that the correlation coefficient value is very low, with only single nodes, and increases rapidly in five hidden layers, finally gradually stabilizing in 10 hidden layers. This is despite the fact that several researchers used only one hidden layer in their ANN architecture, or a small value of hidden layers from 1 to 7 [29,40]. Some studies show that using more hidden layers obtains the best result, such as Campolo et al. [43], who trained the variables with 20, 25, 30, 35, and 40 hidden layers, and Islam [35] as a comparison, with 15, 20, 25, 30, and 35 hidden layers. There are no strict rules for assigning the number of hidden layers and neurons in the literature [21]. The best design for GANN architecture depends on the problem type under investigation. In this study, the GANN model of flood susceptibility used 9 input layers, 10 hidden layers, and 1 output layer.

**Figure 7:**
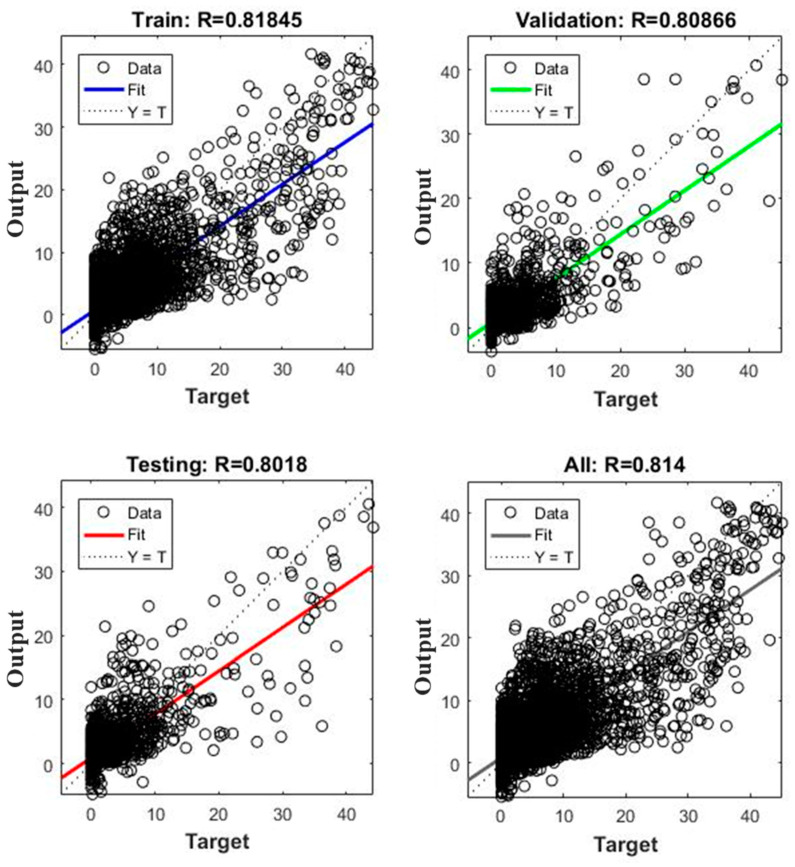
Training process for the correlation coefficient.

The training process reduced the MSE value from 10^3^ to 9.27 in 161 iterations. However, the best performance, at epoch 155, was 9.7189. The performance declined sharply in the first 20 epochs, and then gradually decreased until epoch 161. Figure 7 presents the linear regression for targets relative to the output of the different sub-divisions after the training process was completed.

The efficiency of training the network is represented by the correlation coefficient (R). The cumulative R value was equal to 0.814, which reached the minimum standard of the study requirement (R > 0.8). The training, validation, and testing data sub-division were R = 0.818, 0.808, and 0.801, respectively. The results show that there is a good correlation between the historical flood data and those predicted by the proposed GANN model. According to previous studies [44,45], correlation coefficients whose magnitudes are between 0.7 and 0.9 demonstrate variables which can be considered highly correlated. These results highlight the efficiency of the constructed neural network during the training process and in forecasting flood susceptibility. Our results show that the proposed GANN model could efficiently predict flood susceptibility.

### 3.2. Flood Susceptibility Map

**Figure 8:**
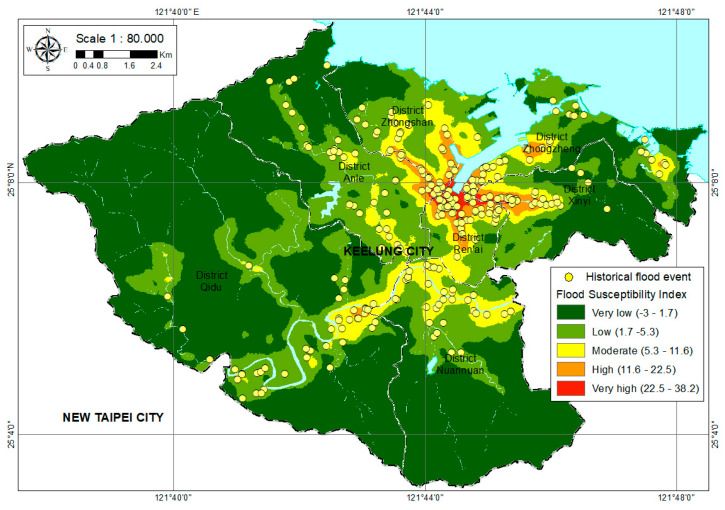
Flood sustainability map of Keelung City.

**Figure 9:**
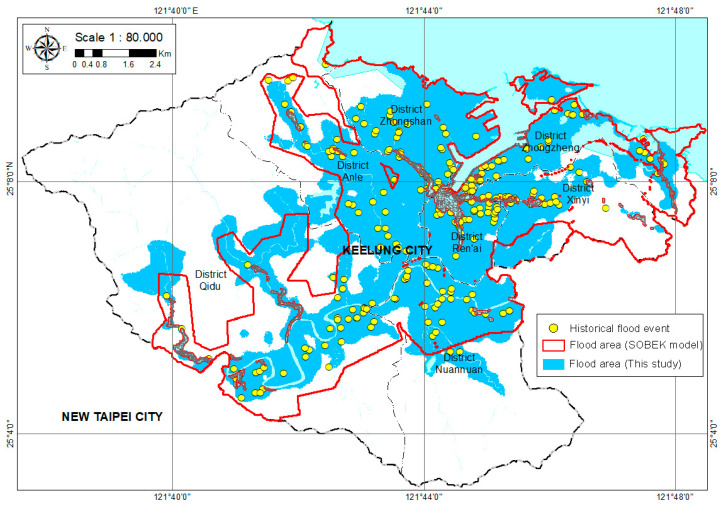
Comparison of the flood area of Keelung City.

The flood susceptibility map is helpful for disaster planning, in addition to being useful during an actual emergency response to floods. Mapping flood susceptibility could be the first step toward mitigating flooding, because flood susceptibility identifies the most vulnerable locations and provides sufficient lead time for an individual to respond to flooding in an anticipatory rather than reactive manner [46]. The computation of the weight of the factors and artificial neural network modeling was performed in MATLAB; the outputs were exported to GIS for map production and visual interpretation. The flood susceptibility map was analyzed qualitatively using natural breaks (jenks) classification schemes [47,48]. The classification of flood susceptibility mapping in Keelung City could be categorized into five classes [23,27,30] based on the value of GANN prediction of the output: Very low, low, moderate, high, and very high. The flood susceptibility index of the five classes is shown in Table 2, and the flood sustainability of Keelung City is displayed in Figure 8. In Figure 8, the results show that a total of 12.1% of the Keelung City area is classified as flood-prone (very high, high, and moderate), according to the GANN model, with a coverage area of 16.14 km^2^. Nearly 3.5% of the study area is in high to very high flood susceptibility zones, as shown in Table 2. The highest susceptibility area was only 1% of the total, and should be prioritized for flood management. To prevent urban inundation in the study, the Water Resources Agency, Ministry of Economic Affairs, ROC has developed a two-dimensional flood forecasting system using the Delft-FEWS platform to integrate the SOBEK models and the precipitation data from the Central Weather Bureau [49]. The flood sustainability obtained from the SOBEK model is adopted for comparison. The comparative results can be seen in Figure 9. It is demonstrated that the overall flood-prone areas from both approaches agree with each other. However, it was also found that the flood sustainability from the SOBEK model was much more conservative than our proposed GANN model. 

The proposed method, based on ANN and GIS, may improve the ability to establish further precise flood models, and present results in a spatial environment. The outcomes of the research could be used to help local authorities to develop appropriate new infrastructure to protect lives and property in Keelung City.

## 4. Discussion

In this research, high elevations occurred in the western part of Qidu District and the southeastern part of Nuannuan District. Meanwhile, the lowland area covered the seashore, especially in the north of Keelung City and the central part, near the Keelung river. The flats area was located in the center of Keelung City, near the flood plain, and a very steep slope is located in the Nuannuan and Qidu District. Based on the GANN results, the most common floods were predicted to occur on the flats, and the northwest (NW), south (S), and southwest (SW) facing slopes. The floods were particularly rare on the north (N) and southeast (SE) facing slopes. Generally, the slopes of the study area are most commonly oriented to the north (N) and southeast (SE) quadrants. To produce a map of the vegetation index using the NDVI method, data from the LANDSAT 8 and high-density vegetation located in the southwest of Qidu District and eastern part of Xinyi District were used. Meanwhile, the non-vegetation area was the built-up area in the downtown region of Keelung City, especially the port area in the Ren’ai District, Xinyi District, Zhongzheng District, Zhongshan District, and southeast of Qidu District. 

Finally, the high and very high prone areas were located in the northern part of the city, as well as in the central part of Keelung City alongside the river. Ren’ai District has the highest susceptibility, followed by Xinyi, Zhongzheng, and Zhongshan District. However, this very high susceptibility area is a core district of the city, which is highly populated, and the economic center of the city. The areas with a classification of very low and low potential coverage were, cumulatively, 87.9% or 116.62 km^2^. These are located at high elevation, especially in the western and southeastern parts of Keelung City, which includes the western part of Qidu and Anle District, as well as the southeast of Nuannuan District.

The novel aspect of this work was to develop a GANN model for flood susceptibility assessment of Keelung, Taiwan. The main contribution of this work is that the proposed method, based on ANN and GIS, may improve the ability to establish further precise flood models, and present results in a spatial environment. The advantages of the GIS spatial analysis capability were integrated into the artificial neural network model. Accordingly, the proposed methodology may represent spatial continuity and the influence of parameters on flood-generating mechanisms. In addition, historical flood data from 2015 to 2019, including 307 flood events, were adopted for the comparison of the flood susceptibility on the regional spatial scale using the GIS. The finding observed in this work may provide a fundamental contribution to environmental protection engineering for flood in areas with higher occurrence and vulnerability to extreme precipitation.

## 5. Conclusions

In this research, the GANN model was developed using 10 flood causative factors. The thematic layers, including elevation, slope angle, slope aspect, flow direction, flow accumulation, TWI, NDVI, and drainage density, were generated using GIS. Rainfall data were also used as input, while historical flood events from 2015 to 2019 were the output of the GANN model.

The proposed GANN model produced satisfactory results, with a coefficient of correlation of 0.81. The susceptibility was categorized into five classes: Very low, low, moderate, high, and very high, with coverage areas of 60.5%, 27.4%, 8.6%, 2.5%, and 1%, respectively. Just 3.5% of the study area was included in the high to very high flood susceptibility zones; however, this area is a core district of the city, with a dense population, and the economic center of the city. 

Furthermore, mapping flood susceptibility is crucial to mitigating flood disasters, since flood susceptibility can identify the most vulnerable areas and predict the potential locations of susceptibility, which can provide authorities responsible for emergency response and evacuation procedures with more information for planning and responses in very high susceptibility areas.

## Figures and Tables

**Table 1:** Source data of the factors and outliers. DEM: Digital elevation model.

Source Data	Factors	Resolution (m)	Maximum Outliers
DEM	Elevation	20	704
DEM	Slope	20	60.40
DEM	Slope aspect	20	358.95
DEM	Flow direction	20	128
DEM	Flow accumulation	20	247,638
DEM	Topographic wetness index (TWI)	20	19.74
Stream river	Drainage density	20	4.07
Rainfall data from the Central Weather Bureau (CWB) of Taiwan	Rainfall interpolation	96	102.65
LANDSAT 8 imagery	Normalized difference vegetation index	30	0.41

**Table 2:** Classification of the flood susceptibility area in Keelung City.

Classification	Flood Susceptibility Index	Area (km^2^)	Percentage (%)
Very low	−3.0–1.7	80.30	60.5
Low	1.7–5.3	36.32	27.4
Moderate	5.3–11.6	11.48	8.6
High	11.6–22.5	3.31	2.5
Very high	22.5–38.2	1.35	1

## Data Availability

The datasets generated during the current study are available from the corresponding authors on reasonable request.
